# The U.S. Travel Health Pharmacists’ Role in a Post-COVID-19 Pandemic Era

**DOI:** 10.3390/pharmacy10050134

**Published:** 2022-10-15

**Authors:** Keri Hurley-Kim, Karina Babish, Eva Chen, Alexis Diaz, Nathan Hahn, Derek Evans, Sheila M. Seed, Karl M. Hess

**Affiliations:** 1 University of California at Irvine School of Pharmacy and Pharmaceutical Sciences, Irvine, CA 92627, USA; 2 Rosary Academy, Fullerton, CA 92831, USA; 3 Canyon High School, Anaheim, CA 92807, USA; 4 Chaminade College Preparatory, West Hills, CA 91304, USA; 5 Portola High School, Irvine, CA 92618, USA; 6 Evans Travel Health, Newport NP18 2NT, UK; 7 Massachusetts College of Pharmacy and Health Sciences, Worcester, MA 01608, USA; 8 Chapman University School of Pharmacy, Irvine, CA 92618, USA

**Keywords:** COVID-19, community pharmacy, travel clinic, vaccines, point-of-care testing, telehealth

## Abstract

**Background:** Many countries have enforced strict regulations on travel since the emergence of the SARS-CoV-2 (COVID-19) pandemic in December 2019. However, with the development of several vaccines and tests to help identify it, international travel has mostly resumed in the United States (US). Community pharmacists have long been highly accessible to the public and are capable of providing travel health services and are in an optimal position to provide COVID-19 patient care services to those who are now starting to travel again. **Objectives:** (1) To discuss how the COVID-19 pandemic has changed the practice of travel health and pharmacist provided travel health services in the US and (2) to discuss the incorporation COVID-19 prevention measures, as well as telehealth and other technologies, into travel health care services. **Methods:** A literature review was undertaken utilizing the following search engines and internet websites: PubMed, Google Scholar, Centers for Disease Control Prevention (CDC), World Health Organization (WHO), and the United States Department of Health and Human Services to identify published articles on pharmacist and pharmacy-based travel health services and patient care in the US during the COVID-19 pandemic. **Results:** The COVID-19 pandemic has changed many country’s entry requirements which may now include COVID-19 vaccination, testing, and/or masking requirements in country. Telehealth and other technological advancements may further aid the practice of travel health by increasing patient access to care. **Conclusions:** Community pharmacists should consider incorporating COVID-19 vaccination and testing services in their travel health practices in order to meet country-specific COVID-19 entry requirements. Further, pharmacists should consider utilizing telehealth and other technologies to increase access to care while further limiting the potential spread and impact of COVID-19.

## 1. Background

The practice of travel health includes providing preventive and self-treatment measures to patients traveling internationally including travel-related immunizations (e.g., yellow fever, Japanese encephalitis, typhoid fever); medications for malaria, traveler’s diarrhea, motion sickness, jet lag, and other diseases; as well as ordering and interpreting laboratory tests for confirmation of vaccine-associated antibodies [1,2]. This is done through a comprehensive review of one’s travel plans such as their destination(s), arrival dates, planned activities, length of stay, and personal health and medical information in order to identify specific travel-related disease risks. Additionally, travel health providers administer appropriate travel-related vaccines and furnish appropriate travel-related prescription and nonprescription medications based off of their patient risk assessment [1]. Organizations such as the CDC and WHO recommend that individuals traveling internationally receive a pre-travel health consultation and any necessary vaccines and medications from a healthcare provider four to eight weeks in advance of their departure [3,4]. It is therefore important that individuals are able to access and receive care in a timely manner so that they can receive all necessary vaccinations and medications prior to their departure.

While pharmacists have traditionally provided travel health services under protocol or through a collaborative practice agreement with a physician, various states and territories in the US now allow for more independent practice [2]. In a review of state laws and regulations, pharmacists in 15 jurisdictions (i.e., a US state or territory) were able to administer all routine vaccines independently and in eight jurisdictions, pharmacists were able to administer all travel-related vaccines independently. Furthermore, in 27 jurisdictions, pharmacists were authorized to furnish travel-related medications to patients and in at least 23 jurisdictions, pharmacists were authorized to order travel health related laboratory tests [1]. Pharmacists intending to practice in travel health should ensure that they have the proper education and training as well as ensure that their clinic area is set up properly with all necessary supplies and is conducive to providing patient care in a separate and private environment but is still adjacent to the rest of workflow [5,6].

The American Pharmacists Association (APhA) has developed the Advanced Competency Pharmacy-Based Travel Health Services Training Program to prepare pharmacists to offer travel health services (https://www.pharmacist.com/Education/Certificate-Training-Programs/Travel-Health (accessed on 13 October 2022)). The successful completion of the APhA Pharmacy-Based Immunization Delivery Certificate Training Program and being an authorized provider of immunizations in one’s state of practice are prerequisites for enrollment in this course which offers 10 h of continuing education through self-study and live seminar components. The certificate program helps prepare pharmacists to evaluate travel itineraries, assess health and safety risks based on destinations, and create and communicate a plan for patients for the necessary prescription and nonprescription medications, immunizations, supplies, and counseling for their trip. This program is based off of the gold standard in travel health knowledge, the Body of Knowledge, which was developed by the International Society of Travel Medicine (ISTM). Furthermore, this Body of Knowledge serves as the basis for ISTM’s Certificate of Knowledge examination for all travel health professionals. Those who successfully complete the exam are awarded the Certificate in Travel Health (CTH^®^) by the ISTM. The CTH^®^ is one of few credentials offered across health disciplines and recognized internationally by health care providers (https://www.istm.org/bodyofknowledge2 (accessed on 13 October 2022)) [5].

Prior to the COVID-19 pandemic, rates of international travel increased annually from 28.5 million outbound international departures (excluding North American destinations) in 2010 to 44.8 million international departures in 2019. In 2020, as COVID-19 began rapidly spreading throughout the world, public health protocols such as limiting international travel began to be enacted to help contain the spread of infection in numerous countries. As a result, international travel dropped to 9.8 million outbound departures in 2020 [4,7]. However, as rates of COVID-19 began to decline and as the pandemic began to evolve into endemicity, international travel has begun to pick back up once again with 26.9 million outbound departures from 2021 to April 2022 [8].

Identifying health risks associated with travel prior to departure has grown in importance as a result of increasing international travel, the dynamic COVID-19 situation, and presence of other infectious diseases more traditionally associated with international travel (e.g., yellow fever, malaria, rabies, etc.). Pharmacists are in an optimal position to provide travel health services given their ability to successfully implement such public health services in community and ambulatory care settings [9,10]. Furthermore, it has been estimated that approximately 90% of the US population lives within five miles of a community pharmacy, thus making community pharmacists arguably the most accessible healthcare provider in the US, allowing more individuals access to care [11]. The extended hours that community pharmacies are open, particularly when other healthcare settings are closed, further improves the public’s access to important public health services such as immunizations and travel health care [12]. During the height of the COVID-19 pandemic, many states also implemented waivers or legislation that allowed pharmacy technicians to administer COVID-19 vaccines to further increase patient access to care. As COVID-19 now transitions to an endemic phase in its epidemiology and many individuals resume pre-pandemic travel schedules, considerations related to COVID-19 will remain a pertinent and dynamic aspect of US travel health care services into the future.

## 2. Objectives and Methods

The aim of this paper is to discuss how the COVID-19 pandemic has changed the practice of travel health, specifically pharmacist provided travel health services in the US, and to discuss the incorporation of COVID-19 prevention practices, telehealth, and use of other technologies into the ongoing practice of travel health care. A literature review from 2018 to the present was undertaken utilizing PubMed and Google Scholar using the following search terms: COVID-19, community pharmacy, community pharmacist, travel health, travel medicine, travel clinic, vaccines, point-of-care testing, and telehealth. In addition, internet websites including, but not limited to, the CDC, WHO, and the US Department of Health and Human Services were identified as other sources of information on pharmacist-managed and pharmacy-based travel health services during the COVID-19 pandemic.

## 3. Results

Thirty-one original research peer-reviewed articles were identified along with information from agency websites listed above that illustrate the impact of the COVID-19 pandemic on travel and healthcare in general. In summary, our literature review shows changes to travel health practices in response to the COVID-19 pandemic including changes in destination country’s entry requirements and regulations, inclusion of COVID-19 counseling and vaccination as part of a travel health services, and the incorporation of telehealth and other technological advancements to the practice of travel health in order to safely (and efficiently) access care. Detailed findings from our literature review are discussed below in the discussion section and grouped according to theme.

## 4. Discussion

### 4.1. Practices and Regulations

As discussed above, individuals who are traveling internationally should contact a travel healthcare provider four to eight weeks before departure to ensure that all necessary vaccines and medications can be provided and administered prior to their trip and that their response to these measures can be monitored [3,4]. This is important since it is also now essential to ensure that travelers are aware of any COVID-19 entry restrictions at their destination while the pharmacist also confirms that they meet that country’s COVID-19 vaccine requirements and perform any necessary COVID-19 testing within the required timeframe. Masking guidelines for public transportation and elsewhere may be specific to destination countries and are also pertinent to patient counseling during travel health visits [13]. Pharmacists that practice in travel health should inform the patient of any restrictions and communicate the required steps for the given destination(s). These procedures are crucial given the ever-evolving and variable nature of COVID-19 waves and public health responses. Various countries often have significantly different requirements and recommendations even where level of risk may be considered similar. Table 1 is included as an example of variation in entry requirements related to COVID-19 in developing countries. It is up to date as of September 2022 but is not intended to serve as a reference for specific travelers or itineraries. Pharmacists who wish to view updated COVID-19 entry information can access this database at the U.S. Department of State’s Bureau of Consular Affairs website at https://travel.state.gov/content/travel/en/traveladvisories/COVID-19-Country-Specific-Information.html [14].

### 4.2. Vaccinations, Testing, and Counseling for COVID-19

#### 4.2.1. Administering COVID-19 Vaccines and Tests as Part of Travel Services

Pharmacists’ administration of COVID-19 vaccines and tests as part of travel clinic services are crucial in protecting the health and welfare of travelers as well as the local communities they visit. The CDC recommends that travelers receive routine vaccinations, including COVID-19 vaccines, to lessen the chance of contracting and spreading infectious diseases [15]. Table 2 provides COVID-19 vaccine resources, as the recommendations and schedules for each vaccine differs depending on age, immunocompetent status, and booster recommendations. Vaccination requirements and recommendations differ based on itinerary, medical history and risk tolerance. Pharmacists providing travel health care services can ensure that vaccines specific to certain countries, including the COVID-19 vaccine, are received in advance [16]. For travel, the CDC has yet to specifically recommend the use of mix-and-match COVID-19 vaccination strategies wherein the formulation of booster dose(s) is different from the primary series. However, this form of vaccination is becoming more common in countries including the U.S. Emerging research has shown that mixed inoculation is beneficial in boosting the effectiveness of vaccinations [17,18]. CDC currently continues to recognize any combinations of accepted COVID-19 vaccines [19].

As of 12 June 2022, COVID-19 tests are no longer required for return entry into the US, but some destination countries may still require testing depending on vaccination status for entry [14]. Providing COVID-19 testing can be a vital service for patients at both pre- and post-travel health appointments. Each country has their own specific testing requirements regarding which test is acceptable and the timing of the test prior to entry. It is best to review the entry requirements of each country during the pre-travel health visit.

Real-Time Polymerase Chain Reaction (RT-PCR) or Nucleic Acid Amplification Tests (NAAT) have a high degree of sensitivity (i.e., correctly identifies disease, produces fewer false negative results) and specificity (i.e., correctly identifies those without disease, produces fewer false positives), detect the RNA of the virus, and are considered the gold standard for COVID-19 identification. Most PCR/NAAT tests are processed in a laboratory setting but some NAAT tests can be performed as a point of care test (POCT) [20]. It is important to note that a false negative result may occur if someone tests too early after an exposure to COVID-19 or if the sample is mishandled or improperly collected.

Antigen-based tests have similar specificity to PCR tests but have less sensitivity, particularly if asymptomatic, they detect a specific viral protein of the virus. Antigen-based tests can be processed in a variety of settings; laboratory, POCT or home self-testing. Antigen tests may need to be performed several times for a definitive diagnosis, a single negative test result is considered a preliminary result and does not rule out illness [20]. Rapid antigen tests were found by other countries to be more inaccurate and less sensitive to detecting COVID-19 than PCR tests [21]. One study concluded that antigen tests misidentified asymptomatic individuals as COVID-19 negative when, in fact, they were carrying the virus [22]. Another study found that the accuracy of rapid antigen tests significantly higher in symptomatic patients rather than in asymptomatic patients, which is most likely due to the higher viral load that could be more easily detected when at the early phases of the disease [23]. However, frequent testing has shown to be useful in detecting the Omicron variant, especially when identifying those with a high enough viral load [24,25]. Rapid antigen tests for this purpose have also been improving in accuracy and sensitivity, but may still produce inaccurate results if the sample is mishandled or improperly collected. In a 2021 study, the Abbott ID NOW test demonstrated a high degree of accuracy and a strong agreement with RT-PCR results, which is similar to the high sensitivity of other tests, such as AQ-TOP and GeneChecker [26]. The current Omicron variant mutations, though, have a portion of the Spike protein (S protein) that cannot be detected by antibodies or targeted by COVID-19 vaccines [27]. These mutations have led to reduced sensitivity in an N-gene or S-gene genetic target with tests, suggesting that a higher viral load would be necessary for rapid tests, which are able to detect multiple genetic targets, to detect the sub-variants [28]. Table 3 includes the similarities and differences between the common types of COVID-19 tests currently available, antibody testing is not used to diagnose a current infection [29,30].

Most community pharmacies in the U.S. administer COVID-19 PCR tests; however, this may pose a significant barrier for travel patients due to the time required for results to be reported since samples are mailed to an outside laboratory for processing. POCT tests such a NAAT test or the Abbott ID NOW test could be utilized in a travel clinic or community pharmacy setting to provide accurate and more timely results for patients.

#### 4.2.2. Incorporating Counseling Regarding COVID-19 into Practice

Providing patient education on COVID-19 rates of transmissibility and preventative measures required by the destination country (masking, quarantine, etc.) is important for pharmacists to consider and implement. Many studies report a lack of general knowledge and/or acceptance regarding the information surrounding the virus and its related vaccinations. A survey carried out among international travelers to non-European destinations in 2021 revealed that approximately 52.4% sourced their information from the internet, while 42.4% sought a doctor or health professional [31]. Another survey of COVID-19 testing offered at US airports led to the conclusion that some travelers might not be able to grasp the meaning of testing “positive” at the airport before taking off, largely because the testing sites are private companies who may be less familiar with advising the public of testing results [32]. In another study, where questionnaires were administered to evaluate participant knowledge on COVID-19 and their perceptions of the COVID-19 vaccine, it was apparent that those without sufficient knowledge about vaccines are more likely to be doubtful towards immunizations for viruses such as COVID-19 [33]. As proven in the data collected from questionnaires and surveys, pharmacist involvement would be vital in preserving post-COVID-19 travel health in a variety of ways, such as through providing advice on safety measures and prevention protocols for travelers [34,35].

Carmosino et al. observed that patient education in promoting increased knowledge and trust in vaccine approval allowed travelers to gain more awareness and have more positive attitudes towards immunizations [36]. Another study found that people with adequate knowledge of COVID-19 were more willing to receive immunizations for vaccine-preventable diseases. Furthermore, 76% of the respondents were willing to receive COVID-19 immunization later on, suggesting that with further guidance and counseling on the pandemic, more people would be open to vaccination [27].

In general, a more informative service regarding infectious disease outbreaks, such as COVID-19, would be helpful to offer at travel clinics. Current trends show that patients are more willing to receive this service from pharmacists. In part with counseling, pharmacists must also work with health care team members to reject misinformation related to COVID-19, vaccinations, and other similar concerns [36].

### 4.3. Telehealth and Other Technologies

#### 4.3.1. Movement towards and Rationale for Distance Consultations

Trends during the COVID-19 pandemic forecast the heavy reliance of pharmacists and patients on technology in the future [37]. Telehealth, or telemedicine, is defined by the US Department of Health and Human Services as “online care provided by a healthcare provider without an in-person office visit” [38]. Prior to the COVID-19 pandemic, telehealth was already being used for patients with inflexible schedules who were unable to attend in-person appointments with their healthcare provider. However, there has been a surge in telehealth implementation during the past two years during the pandemic [37,39,40,41,42].

Healthcare providers offered telehealth as an alternative to in-person scheduled appointments, which allowed continual access to healthcare services which increased as COVID-19 lockdowns prevented individuals from visiting their providers [43]. Some participants in a pharmacy practice workforce survey noted that they faced challenges in adjusting to remote practices at first; also mentioning that in-person contact was still much more valuable [37]. There may also be technical and logistical challenges in implementing telehealth; however, pharmacists should continue working towards expanding telehealth services since information has become highly digitalized in the 21st century and since telehealth can provide added flexibility and is expected to see an increase in its utility in the near future [37]. Table 4 lists various resources and references for pharmacists to consider for implementing telehealth into their practices.

There are various benefits of telehealth to both the patients and providers such as increased access to care, flexible scheduling, decreased transportation costs, and lower cancellation rates [39]. Education and training programs that incorporate resources on telemedicine and remote patient care would be helpful in improving confidence for pharmacists during their assessments [37,43]. Despite these benefits, one of the main concerns with telehealth is that it may limit pharmacists to provide consult-only based services [44]. Overall, telehealth should become a useful addition to a pharmacist’s practice as it allows for ongoing interactions between pharmacists and patients, regardless of hesitancy, for in-person consultations in a post-COVID-19 pandemic world.

#### 4.3.2. Other Technologies/Technological Innovations

Due to telehealth becoming more prominent during the COVID-19 pandemic, other technological advancements need to be made in order to adapt to a changing world. The recent development of smartphone applications has aided in these services with their capability to monitor travel health behavior and warn patients of possible risks at destinations; however, there are some ethical issues such as maintaining up to date information [40]. One such smartphone application is called the Illness Tracking in Travelers (ITIT). ITIT has also been collaborating with the WHO to provide people with prompt public health responses [40,45]. Travel medicine apps can benefit both providers and patients as they can access different types of relevant travel-related data including geolocations, contract tracing, and travel advisories, which would be beneficial in overseeing diseases such as COVID-19.

## 5. Best Practices and Conclusions

The COVID-19 pandemic has increased the strain on existing healthcare systems around the world, necessitating an increased scope of practice for pharmacists. Therefore, in an effort to contain and prevent the further spread of COVID-19 while balancing the increased demand for international travel, various COVID-19 preventative practices should be incorporated into pharmacist provided travel health services. These include COVID-19 patient education and counseling (including methods to reduce one’s risk for COVID-19 and dispelling inaccurate information and myths surrounding COVID-19) as well as incorporating COVID-19 vaccines and testing into practice to help aid the traveler in meeting country entry requirements. Routine immunizations such as influenza and pneumococcal should also be administered for patients regardless of their travel plans in order to ensure that patients are up-to-date with all of their routine vaccines. The advancement of technology and increasing use of remote communications, particularly the use of telehealth during the pandemic, appears to be a lasting trend and its use may continue well beyond the current pandemic. Pharmacists should therefore look to incorporate telehealth practices in their travel health services to increase patient access to care while also minimizing the amount of time that patients would need to be in a health care setting. Limitations to this paper include that it was not undertaken as a systematic review and therefore relevant work may have been excluded which could have strengthened our findings. Furthermore, the COVID-19 situation is rapidly changing, thus potentially rendering certain research or findings out of date. This illustrates the importance that pharmacists need to stay up to date in order to provide the most effective and relevant care to their patients. However, by incorporating COVID-19 prevention measures and adopting telehealth practices, pharmacist provided travel health services can help meet the once again growing demand for international travel while providing their patients with evidence-based recommendations and counseling from the comfort of their own homes.

## Figures and Tables

**Table 1:** Entry requirements for selected popular destinations [14].

Country	COVID-19 Level	Entry Requirements
**Tanzania**	Level 1: Low Risk	Negative COVID-19 laboratory test 24–72 h before departure, completed Traveler’s Health Surveillance Form, and airport health screening which may include on-site rapid antigen testing are all required. COVID-19 vaccination not required. Fully vaccinated travelers are exempt from testing requirements.
**Kenya**	Level 2: Moderate Risk	Negative COVID-19 laboratory test 24–72 h before departure, on-site rapid antigen test on arrival, and completed Traveler’s Health Surveillance Form are all required. COVID-19, vaccination is not required. Fully vaccinated travelers and those under age 5 years are exempt from testing requirements. Masks are required indoors.
**Thailand**	Level 3: High Risk	Full vaccination or negative COVID-19 laboratory test within 72 h before departure is required.
**Brazil**	Level 3: High Risk	COVID-19 vaccination required with limited exception. Negative COVID-19 laboratory test within 24 h before departure is required for unvaccinated travelers. Masks required in certain jurisdictions.
**Argentina**	Level 3: High Risk	Negative COVID-19 test not required, electronic sworn statement required confirming absence of COVID-19 symptoms and vaccination status within 48 h before departure. Medical travel insurance with coverage for COVID-19 related events is required.
**Vietnam**	Level unknown: Risk unknown	Negative COVID-19 test not required, but health screening procedures upon arrival are required.

Note: database last accessed September 2022. Country specific information is subject to change.

**Table 2:** Recommended COVID-19 vaccine information resources for travel health clinicians.

COVID-19 Vaccine Resources		Description
World Health Organization (WHO)—Status of COVID-19 Vaccine within WHO EUL	https://extranet.who.int/pqweb/sites/default/files/documents/Status_COVID_VAX_07July2022.pdf	Guidance document summarizing status of WHO Emergency Use Listing for COVID-19 vaccines, comprehensive of recognized and candidate vaccines worldwide
Centers for Disease Control and Prevention (CDC)—COVID-19 Vaccines	https://www.cdc.gov/coronavirus/2019-ncov/vaccines/stay-up-to-date.html#about-vaccines. https://www.cdc.gov/vaccines/covid-19/index.html	Information for the general public regarding COVID-19 vaccine recommendations in the U.S. COVID-19 vaccine resources for clinicians and public health professionals
Immunization Action Coalition (IAC)—Vaccines COVID-19	https://www.immunize.org/covid-19/	Comprehensive information and numerous communication tools for professionals and patients/general public for U.S. FDA approved COVID-19 vaccines.
Johns Hopkins University Coronavirus Resource Center	https://coronavirus.jhu.edu/vaccines	COVID-19 vaccine information and public health data visualization tools. Includes worldwide country-level data.
American Pharmacists Association (APhA)—COVID-19 Vaccine schedules	https://s3.amazonaws.com/filehost.pharmacist.com/CDN/PDFS/APhA%20Guide%20to%20Vaccination%20Schedules%20Resource%207_27_22_web%20rev.pdf?AWSAccessKeyId=AKIAYICBVAN2V7IWVG4T&Expires=1661624398&Signature=DrhUSbz4t8wTJ2CNGXv3of4inzs%3D. https://www.pharmacist.com/Practice/COVID-19/COVID-19-Vaccines	COVID-19 vaccine resources curated for pharmacists and other clinicians in the U.S. Includes visual COVID-19 vaccination schedules for all ages.
American Health Systems Pharmacist (ASHP)—COVID-19 Vaccines	https://www.ashp.org/covid-19/vaccines?loginreturnUrl=SSOCheckOnly	Clinical, policy, and logistical COVID-19 vaccine information for U.S. health systems providers
Government of Canada—Vaccines for COVID-19	https://www.canada.ca/en/public-health/services/diseases/coronavirus-disease-covid-19/vaccines.html	Information and resources for the general public and professionals regarding COVID-19 vaccines in Canada
National Health System (NHS)—UK Coronavirus (COVID-19) Vaccines	https://www.nhs.uk/conditions/coronavirus-covid-19/coronavirus-vaccination/coronavirus-vaccine/#:~:text=Everyone%20aged%205%20and%20over,dose%20before%20any%20booster%20doses	Information and resources for the general public and professionals regarding COVID-19 vaccines in the U.K., including for international travel.
European Commission—COVID-19 Vaccines	https://ec.europa.eu/info/live-work-travel-eu/coronavirus-response/safe-covid-19-vaccines-europeans_en	Public health, policy, and vaccination strategy information for the European Union.

**Table 3:** NAAT and Antigen Test Differences to Consider When Planning for Diagnostic or Screening Use [20,30].

	Nucleic Acid Amplification Test (NAATs)	Antigen Tests
**Analyte Detected**	Diagnose current infection	Diagnose current infection
**Specimen Type(s)**	Nasal, Nasopharyngeal, Oropharyngeal, Sputum, Saliva	Nasal, Nasopharyngeal, Breath
**Sensitivity (for accuracy)**	Laboratory tests: generally high Point-of-Care tests: moderate-to-high	Generally moderate-to-high at peak viral load. More accurate if symptomatic
**Specificity**	High	High
**Authorized for Use at the Point-of-Care**	Most are not	Most are
**Turnaround Time**	Most are 1–3 days Some rapid tests in 15 min	Most are 15–30 min
**Cost per Test**	Moderate (~$75–$100/test)	Low (~$5–$50/test)
**Advantages**	Most sensitive test available Short turnaround time for NAAT Point-of-Care tests (rare) Usually does not need to be repeated to confirm results	Short turnaround time (~15 min) Allows for rapid identification of infected people, thus preventing further virus transmission in the community, workplace, etc. Comparable performance to NAATs for diagnosis in symptomatic persons and whether a culturable virus is present or not
**Disadvantages**	Longer turnaround time for lab-based tests (1–3 days) Higher cost per test A positive NAAT diagnostic test should not be repeated within 90 days in case detectable RNA is still present after risk of transmission has passed	Less sensitive (more false negative results) compared to NAATs, especially among asymptomatic people and with some variants May need to be repeated to confirm results (any negative test on a symptomatic person should be confirmed with a PCR or NAAT test (CDC, 2022)

**Table 4:** Telehealth/telemedicine resources and guidance.

Reference	Web Link	Summary
World Health Organization WHO—Digital Health	https://www.who.int/health-topics/digital-health#tab=tab_1	Discusses the strategy and resources for use in Digital Health
Health Insurance Portability and Accountability Act of 1996 (HIPAA)	https://www.cdc.gov/phlp/publications/topic/hipaa.html	Federal law creation of standards to protect sensitive healthcare information being disclosed without the patient’s consent or knowledge
ITIT Travel Health	https://www.itit-travelhealth.org	App for illness tracking in travelers
Best Telemedicine Companies in 2022	https://www.healthline.com/health/best-telemedicine-companies#how-we-chose	Options are based upon ratings types of service, pricing accessibility and vetting
Chetu Software Solutions	https://www.chetu.com/healthcare/telehealth.php	Example of a IT company that purpose builds telehealth apps- HIPAA compliant
Itransition—telehealth solutions	https://www.itransition.com/healthcare/telemedicine	Builders of mobile App and web portals—HIPAA compliant
Cogsworth	https://get.cogsworth.com/telemedicine	Advertises they support allied healthcare, clinics and practices- HIPAA compliant

## Data Availability

Not applicable.
